# Polyunsaturated Fatty Acid Imbalance-A Contributor to SARS CoV-2 Disease Severity

**DOI:** 10.1155/jnme/7075883

**Published:** 2025-03-24

**Authors:** James P. Chambers, Luke T. Daum, Bernard P. Arulanandam, James J. Valdes

**Affiliations:** ^1^Department of Molecular Microbiology and Immunology, The University of Texas at San Antonio, San Antonio, Texas 78249, USA; ^2^Lujo BioScience Laboratory, San Antonio, Texas 78209, USA; ^3^Department of Immunology, Tufts University School of Medicine, Boston, Massachusetts 02111, USA; ^4^MSI STEM R&D Consortium, Washington, DC 20036, USA

**Keywords:** dietary imbalance, disease severity, essential polyunsaturated acids, SARS CoV-2, specialized proresolving mediators

## Abstract

**Overview:** SARS CoV-2 infection is accompanied by the development of acute inflammation, resolution of which determines the course of infection and its outcome. If not resolved (brought back to preinjury status), the inflamed state progresses to a severe clinical presentation characterized by uncontrolled cytokine release, systemic inflammation, and in some death. In severe CoV-2 disease, the required balance between protective inflammation and its resolution appears missing, suggesting that the ω-3–derived specialized proresolving mediators (SPMs) needed for resolution are either not present or present at ineffective levels compared to competing ω-6 polyunsaturated fatty acid (PUFA) metabolic derivatives.

**Aim:** To determine whether ω-6 PUFA linoleic acid (LA) metabolites increased in those infected with severe disease compared to uninfected controls.

**Findings:** Increased levels of ω-6 LA metabolites, e.g., arachidonic acid (AA), epoxyeicosatrienoic (EET) acid derivatives of AA (8,9-, 11,12-, and 14,15-EETs), AA-derived hydroxyeicosatetraenoic (HETE) acid, dihydroxylated diols (leukotoxin and isoleukotoxin), and prostaglandin E2 with decreased levels of ω-3–derived inflammation resolving SPMs. Therapeutic treatment of SARS CoV-2 patients with ω-3 PUFA significantly increased 18-HEPE (SPM precursor) and EPA-derived diols (11,12- and 14,15-diHETE), while toxic 9,10- and 12,13-diHOMEs (leukotoxin and iosleukotoxin, respectively) decreased.

**Conclusion:** Unbalanced dietary intake of ω-6/ω-3 PUFAs contributed to SARS CoV-2 disease severity by decreasing ω-3–dependent SPM resolution of inflammation and increasing membrane-associated ferroptotic AA peroxidation.

## 1. Introduction

The two essential diet-derived polyunsaturated fatty acids (PUFAs), linoleic acid (LA) and alpha linolenic acid (ALA), commonly referred to as ω-6 and ω-3 oils, respectively, are 18 carbon long precursors of a number of biologically active PUFA molecules, e.g., eicosanoids (20 carbon long) and docosanoids (22 carbon long).

Crop seeds and vegetable oils are the major dietary sources of LA [1], whereas seafood (wild) is the primary source of ALA [2, 3].  Table 1 shows the desaturase and elongase substrate–product relationships for LA (ω-6) and ALA (ω-3). Since LA and ALA share (i.e., compete as substrates) for the same set of desaturase and elongase enzymes, a significant excess of one can by simple mass action create a conditional deficiency of the other and its downstream products. Thus, the ratio of ingested ω-6 and ω-3 PUFAs in humans is a major determinant of LA and ALA bioconversions and affects the levels of their respective eicosanoid and docosanoid derivatives [4]. It has been demonstrated that altering the dietary ratio of LA to ALA by increasing the concentration of ω-3 PUFA, e.g., to a ratio of 1:1 compared to that of the Western diet ratio (15–16:1), respectively, increases the efficiency of the elongation of ALA to ω-3 derivatives EPA and docosahexaenoic acid (DHA) [4] required for SPM synthesis.

LA is the most abundantly consumed PUFA in the Western diet and the precursor of the two-carbon elongated derivative arachidonic acid (AA), which in humans is the most prevalent PUFA, and a key contributor to the inflammatory process [5, 6]. AA is the precursor for the proinflammatory prostaglandins, leukotrienes, and thromboxanes as well as the proresolving lipoxins. In addition to its being a physiologically active metabolite and precursor, AA is also readily incorporated into cell membrane phospholipids (PLs) (a depot source), which influences membrane-associated receptor function and cell response [7]. Thus, high (nutritionally unbalanced) dietary intake of ω-6 PUFA LA leads to increased levels of AA and incorporation into membrane PLs with far-reaching signaling effects. Considering the need for ω-3 PUFA in the synthesis of specialized proresolving mediators (SPMs) required for resolving inflammation, a dietary imbalance of ω-6 and ω-3 PUFAs can in large part (excluding other nutritional deficiencies and metabolic mutations) account for the inability to resolve SARS CoV-2 infection elicited inflammatory response as well as increased and damaging membrane-associated peroxidation.

The objective of this work was to determine whether a dietary imbalance of the ω-3 and ω-6 essential PUFAs contributed to SARS CoV-2 disease severity. Here, we report that in severe disease, the required balance between protective inflammation and its resolution appears missing. The ω-3 PUFA precursor-derived SPMs needed for resolution are either not present or present at ineffective levels. If dietary PUFA imbalance contributed to disease severity, the restoration of balance by PUFA supplementation should lessen disease severity, which has been demonstrated to be the case. In SARS CoV-2 patients, dietary ω-6 PUFA imbalance is reflected as increased LA metabolites, e.g., AA and its derivatives. Increased AA synthesis promotes not only the generation of physiologically important metabolites but increased incorporation and subsequent peroxidation of membrane-associated AA altering membrane function with far-reaching effects. Such membrane alteration(s) could constitute the “learned” inflammasome-based and diet-induced hyper-responsive immune state, i.e., phenotype proposed by Christ and coworkers [8].

## 2. Inflammation

Inflammation involving the innate and adaptive immune systems is a protective response in which harmful stimuli such as damaged cells, irritants, and pathogens are eliminated, and following inflammation resolution, tissue homeostasis is restored [9]. The SARS CoV-2 infection-inflammation process begins with viral entry into host cells, particularly alveolar cells. Viral induced alveolar cell death, and the accumulation of apoptotic and necrotic cellular debris activates inflammasomes [10], which in turn mediates the secretion of proinflammatory cytokines activating the Phospholipase A_2_ release of AA from immune cell membrane PLs. The released AA is converted to prostaglandins PGD_2_ and PGE_2_ or leukotrienes by immune cell-specific enzymatic activities such as Cyclooxygenase 2 (COX-2) in neutrophils and M1-macrophages, and Lipoxygenase 5 (5-LOX) in neutrophils [11], a process referred to by some as “eicosanoid storm,” i.e., a surge of proinflammatory bioactive mediators including prostaglandins and leukotrienes [12–14]. The eicosanoids subsequently stimulate the production and secretion of cytokines, chemokines, vasoactive amines, and eicosanoids, and their receptor interaction mediated secondary activation of various differentiation programs [15], enhancing the immune response [16–18]. As the inflammatory response progresses, monocytes and lymphocytes accumulate, neutralize harmful substances, undergo apoptosis, and are subsequently cleared by macrophages, thus initiating the resolution process [9].

## 3. Inflammation Resolution

SARS CoV-2 infection-inflammation resolution or lack thereof determines the course of infection and its outcome [15]. To prevent progression from the acute-resolution phase of inflammation to that of persistent-chronic inflammation allowing the restoration of homeostasis, the inflammatory reaction must be actively resolved to prevent further tissue damage [9, 19, 20]. A key event in inflammation resolution is the switching from the synthesis of prostaglandins to that of lipoxins and SPMs [21]. In orchestrated fashion, prostaglandin E2 (PGE2) facilitates the transformation of proinflammatory M1 macrophages to anti-inflammatory M2-macrophages, and upregulation of LOX activity, which mediates the synthesis of SPMs [22]. The switch from M1 to M2 entails changing from the committed synthesis of proinflammatory cytokines such as tumor necrosis factor-α (TNF-α), and interleukins IL-1β and 6 to anti-inflammatory IL-10, respectively [23]. Concomitantly, SPMs are synthesized and function as signals for the termination of the inflammatory response preventing further neutrophil infiltration and secretion of proinflammatory mediators while stimulating macrophage phagocytosing of apoptotic neutrophils and the removal of the pathogen restoring tissue homeostasis [24–27].

The inflammatory response therefore requires a balance between sufficient cytokine production to eliminate the pathogen while avoiding a hyperinflammatory response in which an overabundance of cytokines causes clinically significant collateral damage [28]. If not balanced and left unresolved, proinflammatory components block the return to the uninflamed state and, in the case of SARS CoV-2, leads to the severe clinical presentation characterized by respiratory failure, systemic inflammation, ARDS, multisystem inflammatory syndrome, multiorgan fibrosis, malfunction, and finally to cell death [29]. Because cytokines modulate cross-talk between key enzymes and the immune system during the acute infection event, uncontrolled cytokine release known as cytokine storm results in “excessive” cross-talk and broadening of the metabolic landscape contributing to the biochemical disarray associated with SARS CoV-2 infection progression [30].

### 3.1. PUFA-Derived Resolution Mediators—An Overview

Except for the lipoxin LXA_4_ and DPA_n-6_ resolvins (Rvs), which are ω-6 derived, the resolvins, protectins, and maresins are all ω-3 PUFA derivatives. Lipoxin LXA_4_ is a derivative of AA. Leukocyte-derived 5-LOX catalyzes the synthesis of leukotriene A4 (LTA_4_), which is then converted to LXA_4_ or LXB_4_ by platelet-derived 12-LOX [31]. Collectively, lipoxin LXA_4_, and the resolvins, protectins, and maresins constitute the SPM group. Each SPM is stereospecific exhibiting biological functions, which are cell type and organ specific, and activates in pico-nanogram ranges cell surface G protein–coupled receptors [20, 32, 33]. To date, four human SPM receptors have been identified: ALX/FPR2, ERV1, DRV1, and DRV2, which refer to the ligand used for identification (LXA4, RvE1, RvD1, and RvD2, respectively) although each receptor can interact with additional SPMs [34]. SPMs play a key role in the inflammation resolution process, and macrophages constitute a major source. Phospholipases (sPLA_2_ and cPLA_2_) release PUFAs AA, EPA, DHA, and DPA_n-3_ from membrane PLs from which SPMs are synthesized [35]. Resolvins are resolution phase interaction “products” and inhibit cytokine production, thus “resolving” by controlling leukocyte transportation and removal of inflammatory mediators [14]. Protectins and maresins are macrophage “mediators” in the inflammation resolving process [19, 20, 36–38]. Each is structurally distinct. Although derived via different enzymes, SPMs all share two, and in some cases, three specifically positioned hydroxyl groups arising from peroxy, hydroperoxy, and epoxy precursors.

#### 3.1.1. The E(Eicosanoid)-Series Resolvins (Rvs)

EPA is enzymatically converted by aspirin acetylated COX-2 to 18-hydroxy-eicosapentaenoic acid (18-HEPE, [CH_3_-CH_2_-**C(OH)**H-CH=CH-CH=CH-CH_2_-CH=CH-CH_2_-CH=CH-CH_2_-CH=CH-CH_2_-CH_2_-CH_2_-COO^−^], the precursor for the E-series Rvs [39]. Subsequent hydroxylation by LOX-5 and/or LOX-12/15 gives rise to *Rv*E1 (contains OH groups at positions 5, 12, and 18 [CH_3_-CH_2_-**C(OH)**H-CH=CH-CH=CH-CH_2_-**C(OH)**H-CH=CH-CH=CH-CH=CH-**C(OH)**H-CH_2_-CH_2_-CH_2_-COO^−^]. Both 18-HEPE and *Rv*E1 are anti-inflammatory stopping polymorphonuclear leukocyte (PMN) migration and recruitment to the site of inflammation, thus stimulating resolution [19]. *Rv*E2 contains OH groups at positions 5 and 18 [CH_3_-CH_2_-**C(OH)**H-CH=CH-CH=CH-CH_2_-CH=CH-CH_2_-CH=CH-CH=CH-**C(OH)**H-CH_2_-CH_2_-CH_2_-COO^−^]. *Rv*E3 is the 17, 18 diol [CH_3_-CH_2_-**C(OH)**H-**C(OH)**H-CH=CH=CH-CH-CH=CH-CH_2_-CH=CH-CH_2_-CH=CH-CH_2_-CH_2_-CH_2_-COO^−^]. EPA is also converted by CYP enzymes to epoxy-containing metabolites, e.g., 17,18-epoxyeicosatetraenoic acid (17,18-EpETE).

#### 3.1.2. The D(Docosanoid)-Series Resolvins (*R*v), Protectins (*PD*), and Maresins (*MaR*)

The D-(docosanoid) series constitute two families of LOX/COX-2 hydroxylated molecules that arise from DHA and DPA_n-3_ [39].

##### 3.1.2.1. The DHA-Derived *Resolvins*

These *resolvins* (*Rv*D1-6) are all hydroxylated at position 17 (predominantly *S* configuration) with two containing a second hydroxyl group at either position 3 (*Rv*D6) or 7 (*Rv*D5). Aspirin acetylation of COX-2 produces the *R*-configuration at carbon 17 and is collectively referred to as “aspirin-triggered p*rotectin* and *resolvin* mediators” [20, 40]. The remaining *resolvins* contain a second and third hydroxyl group at positions 7 and 8 (*Rv*D1), 7 and 16 (*Rv*D2), 4 and 11 (*Rv*D3), and 4 and 5 (*Rv*D4). The DHA-derived *protectins* (*PD*1 and *PD*X) are isomeric forms hydroxylated at the 10 and 17 positions. The DHA-derived *maresins* (*MaR*1 and *MaR*2) are both hydroxylated in position 14 with a second hydroxyl group at position 7 (MaR1) or 13 (MaR2), respectively.

##### 3.1.2.2. The DPA_n-3_-Derived *Resolvins*, *Protectins*, and *Maresins*

Docosapentaenoic acid (DPA) consists of two isomers: all *cis*-7,10,13,16,19-docosapentaenoic acid (DPA_n-3_) and all *cis*-4,7,10,13,16-docosapentaenoic acid (DPA_n-6_), and both isomers are abundant in fish oils [41]. Except for the DPA_n-6_-derived resolvin, the *resolvins*, *protectins*, and *maresins* arise from all *cis*-7,10,13,16,19 docosanoid DPA (22:5) isomers. DPA can be generated from both EPA (ω-3 PUFA) and AA (ω-6 PUFA), thus the designation DPA_n-3_ and DPA_n-6_ (*cf*.  Table 1). *Resolvins* (*Rv*D1_n-3_, *Rv*D2_n-3_, and *Rv*D5_n-3_) are all hydroxylated at positions 7 and 17 (*Rv*D5_n-3_) with a third hydroxyl group at position 8 (RvD1_n-3_) or 16 (RvD2_n-3_), respectively. The *protectins* (*PD*1_n-3_ and *PD*2_n-3_) are both hydroxylated in position 17 with a second hydroxyl group at position 10 (*PD*1_n-3_) or 16 (*PD*2_n-3_), respectively. The *maresins* (*MaR*1_n-3_, *MaR*2_n-3_, and *MaR*3_n-3_) are all hydroxylated in position 14 with a second hydroxyl at position 7 (*MaR*1_n-3_), 13 (*MaR*2_n-3_), or 21 (*MaR*3_n-3_), respectively. DPA_n-6_-derived *resolvins* have been observed in fish oils and their inflammatory effects in combination with DHA studied extensively [42–45].

## 4. SARS CoV-2 Infection PUFA Signature

If an imbalance arising from dietary excess of ω-6 LA contributed to SARS CoV-2 disease severity, it should be reflected in those affected with severe disease in a precursor–product manner as increased LA, LA metabolites, and derivatives, e.g., AA and its oxylipin derivatives (prostaglandins, leukotrienes, thromboxanes, and lipoxins), and decreased ω-3 derivatives, e.g., SPMs. LA is one of several cytochrome P450 (CYP)-dependent enzyme substrates and is converted to regiospecific linoleic epoxides (9,10- and 12,13-epoxyoctadecenoic acids) also referred to as leukotoxin and isoleukotoxin, respectively. These epoxides (EpOMEs) are further metabolized by soluble epoxide hydrolase (sEH) to their corresponding diols, i.e., 9,10- and 12,13-dihydroxyoctadecenoic acids (diHOMEs) also referred to as leukotoxin diol and isoleukotoxin diol, respectively.

In six patients with severe SARS CoV-2 infection, McReynolds and coworkers observed plasma epoxide derivatives of LA (EpOMEs) to be significantly elevated (10 times that observed for levels of leukotoxins associated with poor outcomes in ICU-admitted burn patients) as well as elevated levels of diols, i.e., diHOMEs [46]. Also observed in SARS CoV-2 patients and consistent with high levels of ω-6 PUFA LA were increased levels of AA, epoxyeicosatrienoic (EET) acid derivatives of AA (8,9-, 11,12-, and 14,15-EETs), and Prostaglandin E2. Like LA, the EET derivatives of AA are converted into their corresponding diols, e.g., dihydroxyeicosatrienoic acids (DHETs), which are increased in SARS CoV-2 patients [46]. Interestingly, the AA diols were initially thought to be less active than the epoxides; however, the epoxide and diols are now thought to be antagonistic, complicating further the effect of increased levels of AA [47]. The epoxides of ω-3 PUFAs EPA and DHA, epoxyeicosatetraenoic (EEQ) acid and epoxydocosapentaenoic (EDP) acid, respectively, were not detected, nor were the respective diols, i.e., dihydroxyeicosatetraenoic (diHETE) acid and dihydroxydocosapentaenoic (DiHDPE) acid. Paradoxically, a dietary abundance of the LA substrate would be expected to lead to increased biosynthesis of anti-inflammatory epoxides, i.e., EpOMEs. However, following the binding of SARS CoV-2 to the ACE2 receptor, the level of Angiotensin II is increased, which has been shown to induce the expression of sEH [48], resulting in increased conversion of anti-inflammatory EpOMEs to harmful diols, i.e., diHOMEs.

Using serum from 18 moderately severe SARS CoV-2 patients exhibiting respiratory symptoms but not requiring ICU admission, 20 severe patients with respiratory symptoms requiring ICU admission, and 19 healthy subjects without known comorbidities, Schwartz et al. showed the dysregulation of immune regulating lipid mediators [49]. Specifically, increased release of PUFAs from PL plasmalogens suggested differential mobilization and enzymatic hydroxylation of released AA in individuals with moderate and severe disease. As with their diacyl glycerophospholipid counterparts, plasmalogens bear an acyl chain at the *sn*-2 position of the glycerol backbone. The two most common acyl chains in this position are AA and DHA [50, 51]. Moderate disease was characterized by significantly higher levels of the proresolving SPM resolvin RvE3, an EPA derivative. In contrast, severe disease was characterized by a significant increase in AA-derived HETE acid and dihydroxylated diols, i.e., diHETE acid. When grouped according to pathway, moderate disease was characterized by higher levels of COX activity, and LOX catalyzed EPA (ω-3 PUFA) products, whereas severe disease was characterized by increased LOX and CYP enzymatic activities using AA (ω-6 PUFA derivative) as the substrate.

In a study of 38 patients comprised of two groups (critically ill vs. severe disease not requiring mechanical ventilation), Palmas et al. showed that plasma concentrations of SPM resolvins RvD1 and RvE4 were drastically reduced, and diagnostic of disease severity where reduced overall concentrations were observed in those patients who did not survive [52]. A marked increase in the production of AA-derived lipid mediators produced from the essential PUFA LA by both LOX and COX pathways in nonsurvivors compared to survivors was observed, consistent with alterations in lipid mediator biosynthesis, leading to an overall loss in SPM formation, which contributed to a worse outcome in patients with severe disease. Archambault and coworkers conducted a targeted lipidomic analysis of bronchoalveolar lavage from 33 SARS CoV-2 patients requiring mechanical ventilation (2 h following intubation) [53]. At the primary site of infection/inflammation, i.e., the lungs, increased COX metabolites thromboxane and leukotrienes are consistent with increased LA precursor and AA product. Also observed was an increase in LOX epoxide metabolites of LA, AA, EPA, DPA, and DHA. Although epoxides of ω-3–derived EPA, DPA, and DHA increased, suggesting increased EPA, DPA, and DHA conversion to corresponding SPMs, SPMs were not quantitated. Like AA, the required SPM precursors EPA, DPA, and DHA are released from cell membrane PLs for subsequent SPM biosynthesis. Quantitation and assignment of cause and effect is difficult in the absence of the cell source and number of cells. Recruitment and activation of leukocytes to the bronchoalveolar space results in a concentrated debris field comprised of exudates, aggregated platelets, and thrombi (inflammation components) as well as SPMs (resolving components), which collectively appear heterogenous, i.e., an overlap or “coexistence” of inflammation and inflammation resolving processes. It is possible that the observed heterogeneity could arise from differences in the clinical presentation of those studied, such as onset of symptoms and degree of infection severity, resulting in a mixing of acute inflammation events with those of resolution. Nguyen and coworkers observed a significant increase in levels of nonesterified LA and AA PUFAs in the plasma of 27 SARS CoV-2 patients, and the relative proportion of LA among fatty acids was significantly increased in SARS CoV-2 patients, suggesting an alteration of fatty acid metabolism [54].

A recent report suggests that the binding of exogenous LA to a fatty acid binding pocket in the locked structure of the SARS CoV-2 spike protein inhibits virus replication by reducing ACE2 receptor interaction required for cell entry [55]. Phospholipase-released lysophospholipid products are thought to be utilized in the formation of intracellular double membrane vesicles (DMVs), membranous organelle-like replicative structures that serve as sites of viral RNA synthesis [56]. However, the formation of DMVs is a homeostatic equilibrium between free fatty acids and lysophospholipids. Exogenous addition of LA, e.g., 50 μM to cultured SARS CoV-2-infected human epithelial cells, could result in the reacylation of lysophospholipids back to the parent PLs via the Lands cycle [56, 57] decreasing SARS CoV-2 RNA genome equivalents by reducing the number of replicative body DMVs. Thus, binding of LA to the SARS CoV-2 spike protein and reduction of viral replication in cultured cells may be misleading. If LA binding to the spike protein and subsequent inhibition of viral replication is indeed the case in vivo, then LA, which is far more plentiful than other PUFAs in the plasma of SARS CoV-2 patients, should have afforded protection [54].

Free fatty acids (LA, AA, and ALA) have been shown to inactivate enveloped viruses such as Herpes, Influenza, Sendai, and Sindbis [58, 59]. Vivar-Sierra and coworkers conducted an in silico PUFA study to determine their potential to stabilize the SARS CoV-2 spike protein in a “closed” conformation preventing virus binding [60]. PUFAs (ω-3) including DHA and EPA were observed to have more potential in stabilizing the closed conformation in comparison with LA. Goc and coworkers using a spike protein pseudovirus observed that ALA and its elongated derivative EPA blocked the SARS CoV-2 entry [61]. There is evidence indicating EPA and DHA inactivate viruses by causing the leakage or lysis of the viral envelopes [62]. Toelzer has shown the supplementation of infected human cells with exogenous LA synergizes with remdesivir to suppress SARS CoV-2 replication [55]. Although the in vitro effects of LA are interesting, and the supporting experimentation elegant, pause must be taken in proposing LA supplementation. The argument posited here is that long-term, unbalanced dietary intake of ω-6 LA, and its elongation to AA is problematic and leads to increased incorporation into membranes and peroxidation in immune cell membrane PLs. Such alteration impacts signaling, protein trafficking, generation of bioactive lipids, cytokine secretion, and gene activation in both innate and adaptive immune responses [62].

## 5. PUFA Peroxidation

Greater than 80% of hospitalized patients with SARS CoV-2 present with inflammation-driven imbalances of iron homeostasis [63]. Iron sequestration is an important host defense mechanism limiting microbial and viral proliferation and pathogenicity [64]. Ferroptosis, an iron-dependent cell death process, is caused primarily by the accumulation of lipid ROS in cells, resulting in fatal lipid peroxidation [65]. The key chemical modification in PUFA peroxidation is the insertion of oxygen, which can occur both enzymatically and nonenzymatically. The initial event is *bis*-allylic hydrogen atom extraction from a methylene group between two *cis* double bonds of the PUFA generating an alkyl radical. Regardless of how the oxidation is initiated, i.e., by an enzyme such as COX/LOX or non-enzymatic ROS free radical mediated, the initial peroxidation product is always a hydroperoxyl (ROOH) derivative of the PUFA [66].

Iron (Fe^+2^) is essential in driving intracellular lipid peroxidation and ferroptosis. The reaction of Fe^+2^ and H_2_O_2_, known as the Fenton reaction, generates hydroxyl radicals (OH^.^) that remove *bis*-allylic hydrogen atoms from membrane PL PUFAs. This results in the formation of a PL^.^ radical (PL dot), which in turn reacts with O_2_ forming a peroxy (PLOO^.^) radical. A chain of oxidation reactions ensues. The PLOO^.^ removes a *bis*-allylic hydrogen atom from an adjacent PL PUFA to form a hydroperoxyl (PLOOH) derivative and a new PL, which initiates yet another oxidation reaction targeting a neighboring PUFA, protein, and/or nucleic acid [65, 67]. Lipid peroxidation is interrupted by the conversion of hydroperoxides to nontoxic lipid alcohols by glutathione peroxidase 4 (GPX4), which uses glutathione (GSH) as the reducing agent. GSH is a potent cellular antioxidant and the most abundant low molecular weight thiol and plays a crucial role in antioxidant defense against cellular ROS-mediated oxidative damage [68]. Thus, excessive accumulation of iron-dependent ROS results in the depletion of the reducing agent GSH. SARS CoV-2 patients without comorbid conditions were found to exhibit glutamic acid and cystine imbalances [69], two required GSH amino acid precursors. Such imbalances of required GSH precursors suggest an oxidative stress imbalance needing a counterbalancing antioxidant defense, i.e., GSH. GSH levels are correlated with SARS Cov-2 disease severity and lung damage supporting the participation of GSH in disease outcome [70, 71]. A reduction in reactive PUFA PL hydroperoxides to nonreactive and nonlethal PUFA PL alcohols is GSH dependent [70]. Thus, the depletion of GSH by excess generation of ROS has two major effects: inactivation of GPX4, and mobilization of Fe^+2^ for the Fenton reaction, which promotes the propagation of lipid peroxides and ultimately, ferroptosis [72].

Lipidomic studies suggest that membrane components such as the ethanolamine containing glycerophospholipid with *sn*-2 incorporated AA or its 2-carbon elongation product (adrenic acid) are key PL culprits that undergo oxidation (peroxidation) driving cells toward ferroptotic death [56, 72]. PL PUFAs important in the membrane-associated regulation of the inflammation process are the primary substrates for ferroptotic lipid peroxidation [73]. Consistent with the theme that the more the double bonds, the more susceptible [74], exogenous the administration of monounsaturated fatty acid, for example, oleic acid (OA) effectively inhibits erastin-induced ferroptosis by competing with PUFAs for reincorporation/recycling via the Lands cycle (*cf*.  Section 6: Membrane remodeling) into membrane PLs [75]. Importantly, cultured cells supplemented with AA are made sensitive to ferroptosis [76], consistent with the theme that increased ω-6 PUFA leads to increased AA and ferroptosis. Furthermore, PUFA hydroperoxyl derivatives cause ferroptotic death when added to cultured cells with inactivated Glutathione Peroxidase 4 [77]. The remodeling of PUFA-containing membrane glycerophospholipids is key, and decreasing required Lands cycle gene products depletes CoA thioester-activated PUFAs and subsequent insertion into PLs, which are required lipid peroxidation substrates, thus decreasing ferroptosis [72, 77–79]. This further supports the notion that to exhibit lethality after peroxidation, the PUFAs are membrane PL components, and is consistent with affected cell organelle membranes including endoplasmic reticulum, peroxisomes, lysosomes, mitochondria, and Golgi as sites of lipid peroxidation [80].

Initially, enzymatic lipid peroxidation was thought to be carried out mostly by LOXs [76, 77, 81]. Genetic depletion of LOXs protects against erastin-induced ferroptosis [76], supporting the premise that LOXs contribute to ferroptosis. Although free, unesterified PUFAs rather than their esterified PL counterparts have been shown to be the preferred substrates of LOXs [82], Liu and coworkers have recently shown the 12-LOX oxidation of *sn*-2 acyl (AA) in lysophospholipids generated by iPLA_2_ with *sn*-1 specificity [83]. However, other enzymes such as CYP oxidoreductases may also be involved [84, 85]. Yan and coworkers have suggested that two oxidoreductases (NADPH–cytochrome P450, POR, and NADH–cytochrome b5 reductase, CYB5R1) “incidentally” transfer electrons from NAD(P)H to oxygen to generate hydrogen peroxide, which subsequently reacts with iron to generate reactive hydroxyl radicals, resulting in the peroxidation of PL PUFAs during ferroptosis.

SARS CoV-2 infection markedly alters mitochondrial morphology with matrix condensation and swollen cristae consistent with the observed decreased oxidative phosphorylation and inner mitochondrial membrane protein importing, and increased production of mitochondrial ROS [86–88]. Alterations in mitochondrial and endoplasmic membranes could elevate membrane-associated oxidoreductase activity, thus increasing the chance of “incidental” electron transfer to oxygen [84]. Because of the critical role that mitochondria play in cellular energy metabolism, one possible clinical outcome of SARS CoV-2 infection is multiorgan dysfunction arising from reduced nuclear and mitochondrial DNA-encoded oxidative phosphorylation proteins in the heart, lung, kidney, and spleen [89]. Associated with decreased proteins and transcripts of oxidative phosphorylation is increased expression of glycolytic proteins [86, 90–92]. Inhibition of oxidative phosphorylation results in increased mitochondrial ROS generation, which adds to peroxidation ROS damage due to incomplete reduction of O_2_ to H_2_O activating hypoxia-inducing factor (HIF-1α) with a concomitant increase in glycolysis [82]. Thus, such far-reaching metabolic changes could account at least in part for the observed “long” COVID clinical presentation.

## 6. Membrane Remodeling

Importantly, the primary route of AA introduction into membrane PL occurs via the Lands cycle [93]. Following the binding of SARS CoV-2 to the ACE 2 receptor, glycerophospholipids containing esterified LA and AA are significantly perturbed with lysophosphatidyl choline, the predominant PL class (∼60%), and free AA content exhibiting the greatest fold-increase change [56]. Changes in levels of lysophospholipids as well as concomitant increases in released AA, LA, palmitic acid (PA), and OA have been demonstrated in HCoV-229 E-infected host cells [56]. Membrane PLs undergo deacylation–acylation [94, 95] remodeling by the Lands cycle [95], a key connection between the dietary intake of PUFAs and membrane structure, signal transduction, and the immune response. The fatty acid composition of membrane PLs determines the biophysical characteristics of membranes including bilayer bending, membrane curvature, and protein clustering, impacting inflammatory pathways, and responses [96–99].

In the Lands cycle, PUFA is reacylated and reincorporated into lysophospholipids via two enzymes: acyl-CoA synthetase long-chain family member 4 (ACLS) and Lysophosphatidylcholine Acyltransferase 3 (LPCAT3). The major component of cellular membranes is phosphatidylcholine (PC). Although synthesized through the Kennedy pathway, more than 50% of synthesized PC is recycled through the Lands cycle. The acylation of lysophosphatidylcholine by LPCAT3 to produce PC is a key event in the acyl editing process of fatty acid recycling. LPCAT3 prefers as the acceptor lysophosphatidylcholine with a saturated fatty acid at the *sn*-1 position and *sn*-2 PUFA-CoA donor preference of LA and AA. Importantly, LPCAT3 has been shown to impact cell function by remodeling membrane PLs during inflammation and regulating endoplasmic reticulum membrane stress-inflammation response [99]. AA being a preferred substrate for ACLS [100] and LPCAT3 makes AA a significant competing substrate of EPA and DHA and contributor in the reacylation of membrane PLs [101].

Direct oxidation by COX/LOX enzymes or ROS gives rise to a variety of reactive oxidized PLs that can affect membrane integrity and function. Alternatively, PLA_2_ activation and release of AA from PLs can be subsequently oxidized in a similar fashion giving rise to peroxidation intermediates (peroxyl and hydroperoxyl) and hydroxylated PUFA products, which are subsequently re-esterified via Lands cycle enzymes back into membrane lysophospholipids. Importantly, incorporation of hydroxylated acyl chain lipids into cellular PLs has been shown to alter membrane bilayer molecular dynamics, thus changing the physical properties of membrane-associated function(s) [102]. Furthermore, oxidized PLs have emerged as potent signaling molecules in many systems [103]. Upregulation of membrane-associated protein functions by enzymatically modified PUFAs may represent, as suggested by Spickett, the “epilipidome” analogous to that of the epigenetic modifications that control gene expression [104]. Such complex and far-reaching interaction is further underscored by the novel concept that membranes may serve as allosteric ligands that can modulate enzymes, e.g., PLA_2_ enzyme specificity via *sn*-2 positioning of PL molecules affecting hydrophobic binding and recognition [105].

## 7. PUFA Supplementation in SARS CoV-2 Patients

Nutritional deficiencies influence both immune response and viral pathogenic functions [106, 107] and have been shown to drive the development of a range of inflammatory diseases [108]. Consistent with the resolution of the initial acute inflammatory response, higher levels of ω-3–derived SPMs are linked with favorable outcomes in severe SARS CoV-2 disease [52]. Dysregulation of plasma lipid mediator profiles in critically ill SARS CoV-2 patients has been observed, providing a clear link between disease severity and survival outcome with the plasma concentration of the SPMs RvD1 and RvE4 [52]. SPMs have been shown to be protective, promoting the resolution of acute lung lesions in acute respiratory distress syndrome (ARDS) [109]. Doaei et al. demonstrated ω-3 PUFA supplementation improved the levels of several parameters of respiratory and renal function in critically ill SARS CoV-2 patients [110]. Sobrino et al. using human macrophages from healthy volunteers have shown that augmentation with ω-3–enriched oils upregulated SPMs, suggesting that the restoration of SPM concentrations represents an alternative therapy for vascular inflammation [111]. Arnardottir and coworkers present data supporting SPM-induced resolution of inflammation by the ω-3 PUFA treatment of SARS CoV-2 patients with a significant increase in plasma EPA, DHA, and the critically important PUFA SPM precursor 18-HEPE while EPA-derived diols 11,12- and 14,15-diHETE increased, toxic LA-derived leukotoxin diols 9,10-diHOME decreased [112]. Furthermore, the uncontrolled SARS CoV-2 inflammatory response was significantly reduced as evidenced by the reduction in the ratio of neutrophils to lymphocytes [112]. Pawelzik and coworkers have demonstrated decreased oxidative stress and beneficial alteration in the urinary oxylipidome following intravenous ω-3 administration in hospitalized SARS CoV-2 patients [113]. The above observations are all consistent with and supportive of the 2018 recommended use of ω-3 PUFA–rich fish oil in enteral and parenteral nutrition due to its anti-inflammatory and immune-modulating effects: reduction in the infection rate, and length of hospital stay in medical and surgical patients admitted to the ICU [114].

## 8. Conclusions

**Figure 1:**
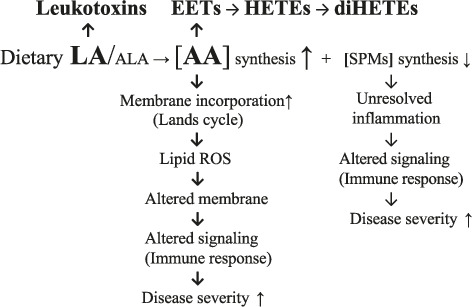
Summary of unbalanced dietary PUFA intake and SARS CoV-2 PUFA signature. LA, ω-6 linoleic acid; ALA, ω-3 α-linolenic acid; SPMs, specialized proresolving mediators; ROS, reactive oxygen species (peroxidation products); AA, arachidonic acid; EET, epoxyeicosatrienoic; HETE, hydroxyeicosatetraenoic acid; diHETE, dihydroxyeicosatetraenoic acid; , concentration.

Although the number of SARS CoV-2 patients studied is small compared to the total number of cases reported, available data reveal an increased ω-6 PUFA metabolic contribution in those exhibiting more severe disease. As summarized in Figure 1 and for the reasons previously given, unbalanced (LA) dietary PUFA intake gives rise to (1) deficient ALA-dependent SPM synthesis and unresolved inflammation, and (2) increased LA-dependent generation of cytotoxic leukotoxins and diols (diHOMEs), AA metabolites (EETs, HETEs, and diHETEs), and AA membrane-associated ROS peroxidation.

Like other phospholipase-released PL PUFAs, e.g., SPM precursors EPA and DHA, the much more abundant AA also serves in a competitive fashion as the substrate for COX, LOX, and Cytochrome P450 (CYP) enzymes compromising SPM synthesis. Furthermore, LA and ALA share (i.e., compete) as substrates the same set of desaturase and elongase enzymes. A significant excess of one can by simple mass action create a conditional deficiency of the other and its downstream products, e.g., SPMs. In humans, a major determinant of LA and ALA bioconversions affecting the levels of their respective eicosanoid and docosanoid derivatives is the ratio of ingested ω-6 and ω-3 PUFAs [4]. Alteration of the dietary ratio of LA to ALA by increasing the concentration of ω-3 PUFA, e.g., ALA to a ratio of 1:1 increases the efficiency of elongation of ALA to ω-3 derivatives EPA and DHA [4] required for SPM synthesis and inflammation resolution. If dietary PUFA imbalance contributed to SARS CoV-2 disease severity, restoration of balance by PUFA supplementation should lessen disease severity consistent with observations by a number of investigators. Interestingly, LA was once considered a rare dietary lipid. However, LA from vegetable oil (in particular soybean oil, which is approximately 55% LA) is now a major lipid in the Western diet.

Christ and coworkers using an atherosclerosis mouse model have shown that the immune system misinterprets the Western diet as a threat to the host and sets in motion powerful anti-infectious mechanisms [8]. The Western diet, which is deficient in ω-3 PUFA with excessive amounts of ω-6 PUFA (15–16/1), is very different than that diet on which humans evolved (approximately 1.0), and their genetic patterns established [115]. Mechanistically, the NLRP3 inflammasome was identified as the central receptor, which mediates diet-induced systemic inflammation and myeloid precursor reprogramming resulting in a hyper-responsive state with generation of myeloid cells that are programmed to respond to secondary inflammatory triggers more strongly. Prolonged and excessive incorporation of AA into membranes and its subsequent peroxidation may damage hematopoietic stem cell and bone marrow membrane microenvironments. Thus, the alteration of the myeloid cell membrane could give rise to myeloid cell reprogramming and a long-lasting hyper-responsive diet-induced phenotype. Given the multigenic and multifactorial nature of chronic inflammation in general, the optimal ω-6/ω-3 ratio may vary considerably due to metabolic predispositions [115].

The nutritional basis of the linkage between inflammation resolving ω-3 PUFAs and clinical outcome is complex, but a clear association exists albeit many questions remain unanswered. However, the critical need for additional studies to answer the many unanswered questions cannot overshadow the known need for and potential beneficial effects of ω-3 PUFAs in resolving inflammation and in the case of SARS CoV-2 reducing disease severity. In addition to the immense costs attributed to the SARS CoV-2 pandemic of 2019 in terms of lives lost, social upheaval, and the severe taxation of the health care delivery system, the CDC estimates over 440,000 deaths secondary to SARS CoV-2 infection in the United States, now a leading cause of morbidity and mortality [116]. Although our understanding of the many metabolic interactions and connections following SARS CoV-2 infection is incomplete, a simple but measured strategy of using nutritionally balanced essential PUFA dietary supplementation (based upon that which is known) constitutes an inexpensive alternative protective approach.

## Figures and Tables

**Table 1:** Summary of metabolic substrate–product relationships for ω-6 and ω-3 PUFAs.

ϖ-6	ϖ-3
LA (18:2)	ALA (18:3)
↓	↓
GLA (18:3)	SA (18:4)
↓	↓
DGLA (20:3)	ETA (20:4)
↓	↓
AA (20:4)	EPA (20:5)
↓	↓
22:4	DPA (22:5)
↓	↓
24:4	24:5
↓	↓
24:5	24:6
↓	↓
DPA (22:5)	DHA (22:6)

Abbreviations: AA, arachidonic acid; ALA, α-linolenic acid; DGLA, dihomo-γ-linoleic acid; DHA, docosahexaenoic acid; DPA, docosapentaenoic acid; EPA, eicosapentaenoic acid; ETA, eicosatetraenoic acid; GLA, γ-linoleic acid; LA, linoleic acid; SA, stearidonic acid.

## Data Availability

The authors confirm that the data supporting the findings of this review are available per the literature cited. Data sharing is not applicable as no new data were created.

## Nomenclature

AA: Arachidonic acid
ACE2: Angiotensin cleaving enzyme 2
ACLS4: Acyl-CoA synthetase long-chain family member 4
ALOX: Arachidonic acid lipoxygenase
ALA: Alpha linolenic acid (ω-3)
ARDS: Acute respiratory distress syndrome
cPLA_2_: Cytosolic phospholipase A_2_
CoA: Enzyme CoA
COX: Cyclooxygenase
CYB5R1: NADH cytochrome b5 reductase
CYP: Cytochrome P450 oxidase
D: Docosanoid
DGLA: Dihomo-γ-linoleic acid
DHA: Docosahexaenoic acid
DHET: Dihydroxyeicosatrienoic acid
DiHOME: Diyhdroxyoctadecenoic acid
DMV: Double membrane vesicle
DPA: Docosapentaenoic acid
DTA: Docosatetraenoic acid
EET: Epoxyeicosatrienoic
EPA: Eicosapentaenoic acid
EpOME: Epoxyoctadecenoic acid
FADS2: Fatty acyl desaturase 2
GPX4: Gluthione peroxidase
GSH: Glutathione (reduced)
HETE: Hydroxyeicosatetraenoic acid
diHETE: Dihydroxyeicosatetraenoic acid
LA: Linoleic acid (ω-6)
LPCAT3: Lysophosphatidylcholine acyltransferase 3
LOX: Lipoxygenase
LXA4: Lipotoxin A4
MaR: Maresin
NADPH: Nicotinamide adenine dinucleotide phosphate (reduced)
NLRP3: Nucleotide-binding domain, leucine-rich-containing family, pyrin domain-containing-3
OA: Oleic acid
PC: Phosphatidylcholine
PD: Protectin
PG: Prostaglandin
PL: Phospholipid
PMN: Polymorphonuclear
POR: NADPH cytochrome P450 oxidoreductase
PUFA: Polyunsaturated fatty acid
ROS: Reactive oxygen species
Rv: Resolvin
SARS CoV 2: Severe acute respiratory syndrome coronavirus-2
sEh: Soluble epoxide hydrolase
*sn*: Nucleophilic substitution
sPLA_2_: Secreted phospholipase A_2_
SPM: Specialized proresolving mediator
TNFα: Tumor necrosis factor alpha
TXA2: Thromboxane A2
